# Medicines prescribed in pregnancy: Protocol for a signal detection study using routinely collected data in England

**DOI:** 10.1371/journal.pone.0349045

**Published:** 2026-05-26

**Authors:** Liza Bowen, Hannah Johnson, Iain M. Carey, Tess Harris, Emma Baker, Derek G. Cook, Pensee Wu, Michael R. Perkin, Alex Barr, Joan Morris

**Affiliations:** 1 Department of Population Health and Policy, City St George’s University of London, London, United Kingdom; 2 School of Medicine, Keele University, Keele, United Kingdom; 3 Public contributor; PLOS: Public Library of Science, UNITED STATES OF AMERICA

## Abstract

**Introduction:**

Medication use in pregnancy is common and increasing. Pregnant women are often excluded from drug trials, therefore the safety to mother and baby of many medications used in pregnancy is unknown. Routine health data have been used to look for evidence of harm and safety of specific medications, but not to systematically look across all prescribed medications in pregnancy.

**Aims:**

To systematically identify medications prescribed during pregnancy requiring further research into their potential harms to mother or baby.

**Methods:**

We will describe trends in primary care prescribing in pregnancy over time, and socio-demographic patterning of prescribing, using data from pregnancies in the Clinical Practice Research Datalink (CPRD). We will identify and categorise maternal, fetal, and infant adverse outcomes reported in the CPRD and linked hospital and mortality data. We will use Bayesian signal detection models to analyse all medication/outcome pairs to identify where an outcome occurs significantly more frequently in mothers prescribed a specific medication than in mothers not prescribed it. Potential confounders will not be accounted for at this stage. Published evidence on the identified medication/outcome pairs will be reviewed and incorporated with the results from trends and signal detection analyses. These data will be used by Patient Involvement and Study Advisory Board workshops to prioritise medications for further research, with medications more commonly prescribed for underserved groups being given precedence. For three prioritised signals, cohort analyses will compare the occurrence of adverse outcomes in those prescribed compared to those not prescribed the medication of interest, adjusting for maternal morbidity, co-medications, and other measured confounders.

**Discussion:**

This project will provide a list of medications used in pregnancy that should be prioritised for further safety research. Results of three detailed retrospective cohort studies will contribute to informed decision making for patients and clinicians. Study grant number: NIHR207172; CPRD accepted protocol number: 24_004518.

## Introduction

Medication use in pregnancy is common and increasing [1–4]. However, randomised controlled trial evidence on medication safety (to mother and baby) is lacking because pregnant women are often excluded from clinical trials [5]. Using routine databases is one way that we can begin to fill the research gap that is left from the exclusion of pregnant women from drug trials [6,7]. Safety of medications in pregnancy emerged as a top priority of both the public and stakeholders (clinicians, researchers, charities) in a recent review of pregnancy research [8], and in the 2023 UK Women’s health strategy [5].

Changing socio-demographic and health profiles mean that UK women are now having pregnancies at older ages with more comorbidities, and are therefore more likely to take medications during pregnancy [9]. Some studies estimate that over 80% of women in high income countries take a prescribed medicine during their pregnancy [1,2,9,10]. There are also important changes in prescribing practices. Anti-depressant use in women of childbearing age has been increasing year on year in the UK, from 2.1 million women aged 15–49 in 2016 to 2.6 million in 2024 [11]; this has implications for the number of women exposed in pregnancy and requires further investigation [11–13]. Understanding the variations in medication use in pregnancy over time, and between different socio-demographic groups, can highlight important factors that could contribute to bias and confounding. It is therefore important that descriptive analyses of trends precede subsequent drug safety studies.

There is a growing number of medications that would benefit from greater information on safety of use in pregnancy. Once a concern has been raised about a medication that is being prescribed in pregnancy being potentially harmful, studies can be undertaken to evaluate the harm. However, to our knowledge, there are no studies that are aimed at systematically identifying potential maternal, fetal, and infant harms of medications in pregnancy, which could then be prioritised for further research into their safety. This is important when so many of the medications used in pregnancy have insufficient safety data. Signal detection is the process of looking at pattens of adverse outcomes within datasets to identify potentially causal associations between a medication and the adverse outcome. Statistical methods are available that allow for analysing a large number of medications and outcomes at once, identifying a small number of medications where an outcome is occurring more often than would be expected from chance alone. These approaches are well established in general pharmacovigilance but have rarely been applied to the full range of maternal, fetal, and infant outcomes in pregnancy. Applying such methods to linked primary care, hospital, and mortality records provides an opportunity to identify previously unrecognised safety signals. This in turn enables us to prioritise medications that need further research into their safety.

This project will investigate safety of medications by evaluating a wide range of potential adverse outcomes for mother, fetus and infant based on the set of core data elements for pregnancy pharmacovigilance developed by the European Network of Teratology Information Services and including maternal and infant mortality, maternal medical conditions arising in pregnancy, delivery and post-partum complications, fetal loss and neonatal and infant morbidity [14].

## Materials and methods

The overarching aim of this study is to identify medications that may be harmful to the mother, fetus, or infant, when prescribed during pregnancy.

The specific objectives are:

1. To describe temporal trends in medications prescribed during pregnancy in primary care in England over the past 20 years, and to examine variation by maternal age, ethnicity, socio-economic status, geographical region, and comorbidities.
2. To apply Bayesian signal detection methods to identify medications potentially associated with adverse maternal, fetal, or infant outcomes.
3. To integrate signal detection results with existing scientific evidence and expert and patient input in order to prioritise medications requiring further investigation.
4. To conduct detailed retrospective cohort studies of three prioritised medications to quantify associations with adverse outcomes after adjusting for confounding.

### Data source

**Fig 1:**
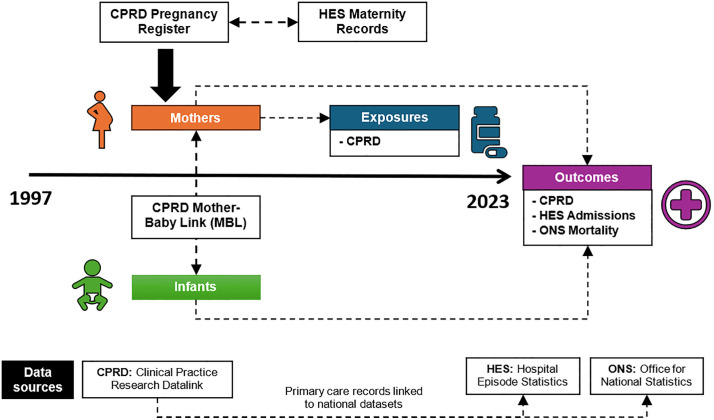
Study population and data sources for exposures and outcomes.

This is a population-based observational study using routinely collected electronic health records from the Clinical Practice Research Datalink (CPRD) Gold and Aurum databases. Pregnancy episodes will be identified using the CPRD Pregnancy Register, with linkage to Hospital Episode Statistics (HES), Office for National Statistics (ONS) mortality records, Index of Multiple Deprivation (IMD) data, and the Mother–Baby linkage for ascertainment of infant outcomes (Fig 1).

We will use data from the CPRD pregnancy register to identify pregnancy episodes and estimated delivery dates and the Mother-Baby link to identify infant outcomes [15,16]. CPRD contains data contributed by GP practices using Vision (Gold) and EMIS (Aurum) clinical systems. The CPRD pregnancy register has been validated against linked HES and the ONS live birth data [15]. Data will be linked to HES data, ONS death registration data, and IMD data.

The study protocol was approved by CPRD’s Research Data Governance group (Ref: 24_004518). Based on these approvals individual patient consent was not required.

Analyses will be conducted within CPRD Safe (trusted research environment).

### Study population

The study will include women in the CPRD pregnancy register who have been registered for at least six months before the start of each pregnancy to ensure sufficient recording of long-term health conditions. An individual woman can contribute more than one pregnancy. The CPRD pregnancy register uses an algorithm based on primary care records to generate information on likely pregnancies, dates, and outcomes. This pregnancy register has been validated and can be supplemented with hospital admissions data to further improve accuracy of information on pregnancy dates and outcomes [15,17,18].

Non-singleton pregnancies will be excluded. Mother and baby will be followed up for up to one year after birth to look at maternal post-partum and infant outcomes.

### Exposures

Data on all primary care prescriptions from 90 days prior to pregnancy through to the end of pregnancy, including those that have been initiated in secondary care and continued in primary care, will be extracted. Prescribing patterns will be assessed throughout pregnancy and by trimester and 90 days prior to pregnancy start. Trimesters will be defined as per the CPRD pregnancy register. Medications will be classified using British National Formulary (BNF) and Anatomical Therapeutic Chemical (ATC) systems.

### Outcomes

Adverse outcomes will be identified using coded data from primary care, hospital admissions, and linked datasets. Outcomes will be grouped into maternal, fetal, and infant categories, based on the Core Data Elements for pregnancy pharmacovigilance developed by the European Network of Teratology Information Services and ConcePTION consortium [6,14,19] (Table 1).

**Table 1:** Outcome groups to be extracted from health records.

**Maternal**	Maternal medical conditions arising in pregnancy
	Maternal delivery complications
	Maternal post-partum complications
	Maternal death
**Fetal**	Molar pregnancy
	Ectopic pregnancy
	Miscarriage
	Premature delivery
	Congenital anomaly
	Stillbirth
**Infant**	Small-for-gestational-age at delivery
	Large-for-gestational-age at delivery
	Neonatal death
	Infant death
	Hospital admissions

The maternal medical conditions arising in pregnancy outcomes will include: gestational diabetes; hypertensive disorders of pregnancy; venous thrombotic episode; depression/anxiety/psychosis; pulmonary oedema; acute kidney injury; stroke; maternal infections; antepartum haemorrhage. The congenital anomaly outcome will include major congenital anomaly subgroups of heart, limb, kidney and urinary tract, digestive system, nervous system, abdominal wall, respiratory, and oro-facial clefts.

For our clinical outcomes, some of the relevant code lists have already been developed and will be accessed from code repositories and publications and reviewed by our clinical team, e.g., pre-eclampsia [20], stroke [21], congenital anomalies [22]. Some pregnancy related outcomes are also included in the pregnancy register data received from CPRD, e.g., stillbirth, miscarriage, ectopic pregnancy, molar pregnancy [15]. For outcomes with no appropriate published codelists, we will develop new code lists.

### Covariates

Ethnicity will be based on self-reported ethnicity recorded in HES, or where HES ethnicity is missing, the most recent recorded ethnicity in primary care records. Socio-economic status will be measured by the linked Index of Multiple Deprivation data. Geographic regions will be defined as the Office of National Statistics (ONS) regions assigned to each practice by CPRD. Comorbidities (e.g., asthma, diabetes, migraine, depression/anxiety) at the start of pregnancy will be extracted from CPRD and HES codes.

## Statistical analysis

### Objective 1: Descriptive analysis

Individual medications will be grouped into summary categories broadly based on BNF chapter and section (S1 Table) [23]. Binary variables will be created for whether a medication from each of the categories was prescribed at any time during the pregnancy, each trimester of pregnancy individually, and for the three-month period prior to the start of pregnancy. Rates of prescribing of each medication category will be calculated per 1000 pregnancies.

To look at trends over time, rates of prescribing will be calculated for each year separately, with a pregnancy assigned to a single year based on the start date of the pregnancy.

Factors influencing the prevalence of prescribing of medication groups during pregnancy will be investigated by modelling whether a prescription was issued for a given medication group during pregnancy, using multivariable logistic regression models including variables such as maternal age, ethnicity, geographical region, deprivation, and comorbidities.

### Objective 2: Signal detection

Adverse outcomes will be defined as above. Exposures will be prescriptions of individual active ingredients of medications. The estimated dates of pregnancy will be used to identify any medications prescribed in the three months prior to pregnancy or during pregnancy, and analyses will be run for each trimester separately as well as for the three-month period prior to pregnancy.

We will apply standard Bayesian signal detection models using the Information Component (IC) [24–26]. This method is based on comparing the frequency of occurrence of the outcome of interest in mothers prescribed the medication of interest compared to the frequency of occurrence of the outcome of interest in mothers not prescribed the medication of interest for each medication/outcome pair. IC values that are positive indicate that the outcome and prescribed medication occur more frequently together than would be expected due to chance if both events were independent. In brief the observed counts (a,b,c,d from a two-by-two table) are assumed to have a Multinomial distribution (pa,pb,pc,pd,n). With a Dirichlet prior distribution for pa pb pc and pd the corresponding posterior distribution is also a Dirichlet distribution. Assuming an uninformative prior likelihood, 100,000 random samples from the proposed posterior Dirichlet distribution can be generated and the median IC with its 95% credible intervals obtained [27]. Pairs with a positive lower 95% credible interval have sufficient evidence that they are occurring more frequently than would be expected due to chance and are classified as signals.

Our new method of hierarchical modelling will then use these results to identify groups in the data using bi-clustering techniques. For this method, pairs with a positive lower 90% credible interval using the IC are considered ‘potential signals’. A group is defined as containing all medication/outcome pairs where all medications in the group have a potential signal with the same outcomes in the group. Medications with similar pharmacological properties are likely to be in the same groups, but the actual group membership is determined purely by the data. These groups will then be used to calculate an observed to expected ratio, adjusted for groupings, for each medication/outcome pair.


$$\begin{array}{c} Log(Proportional\;Reporting\;Ratio\;\left(PRR)\right)=\;\frac{Observed\;Frequency\;of\;Outcome\;and\;Medication}{Expected\;Frequency\;of\;Outcome\;and\;Medication} \end{array}$$

As with the IC, values that are positive indicate that the outcome and exposure occur more frequently together than would be expected due to chance if both events were independent [24,25].

The log(observed/expected) for each medication/outcome is assumed to have a gaussian distribution where the mean has two components – one that is determined by the mean of all medications and outcomes in the same group and an additional component specific to each medication/outcome. This assumes that similar medications may have similar safety profiles and will result in all the medication/outcomes in a specific group having more similar results than if the Log(observed/expected) was calculated independently for each pair. As with the IC, the posterior distribution is derived from the combination of the prior and the likelihood from the observed data. However, as the posterior distribution is not the same as the prior distribution Monte Carlo Methods need to be employed to derive it. Any medication/outcome pairs with the 95% credible interval above 1 will be interpreted as generated signals.

### Objective 3: Prioritisation process

**Fig 2:**
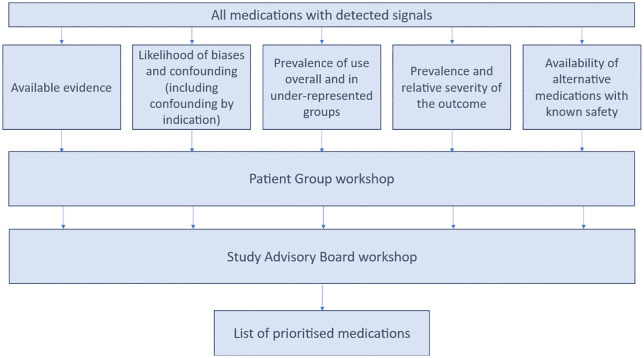
Process for selecting medications for detailed analysis.

Once signals have been generated, our next objective will be to combine these signals with descriptive information on prescribing (objective 1), current available evidence, and workshop discussions with a multidisciplinary group of clinicians, epidemiologists, and patient and public involvement representatives. Information from the following domains will be considered when prioritising medications(Fig 2).

1. **Available evidence**

For all signals identified we will annotate them with information from the BNF, UKTIS, Reprotox, TOXBASE and DART. We will add information from searches on PubMed and Ovid.

Each individual medication-outcome pair will be annotated as

i known harmful effect
ii known indication effect (association between the reason the medication is taken and the outcome)
iii unknown – limited evidence for specific pair
iv unknown – limited evidence for composite exposure (broad medication groupings) and/or composite outcome (grouping of specific outcomes)
v unknown – no evidence

1. **Likelihood of biases and confounding (including confounding by indication)**

Information on whether the medication is available over the counter will also be included because this can result in bias due to misclassification of exposure. An initial assessment of likelihood of confounding by indication will be discussed with input from clinicians. We will check for frequency of co-medication. This will highlight any signals that may be due to confounding by co-medication if they are prescribed >50% of the time with a medication that has a known effect on the relevant outcome.

1. **Prevalence of use overall and in under-represented groups**

Commonly prescribed medications will be given priority over those prescribed more infrequently. We will also be able to identify any medications prescribed more frequently in under-represented socio-demographic groups from our results in objective 1, which will be an additional priority criterion.

1. **Prevalence and relative severity of the outcome**

Initial assessment of severity of outcome will be done by the study team, but Patient and Public Involvement (PPI) input on this will be vital to ensure that their priorities feed into an understanding of which outcomes are most important to women with lived experience of patient decision making around medication use in pregnancy. The magnitude of the estimated increased risk of the outcome together with the prevalence of the outcome will be summarised as population attributable risks (i.e., the proportion of cases of the relevant adverse outcome that would be attributed to the use of the given drug in pregnancy if the association were causal).

1. **Availability of alternative medications with known safety**

The potential for change in prescribing practice is greater for medications where there are alternative medications that can be prescribed for any given indication. In cases where there are not known safe alternatives the risks of not taking a given medication will be an important consideration.

Initial collation of information gathered from the above 5 domains will be done by the study team and formatted in an appropriate way for an initial PPI workshop. Output from this workshop will be added to the information from the initial 5 domains and taken to the Study Advisory Board Workshop. The multidisciplinary team will review information from the 5 domains and PPI discussions prior to the workshop and during the workshop will have small group and whole group discussions through which a prioritised list of medications will be generated.

### Objective 4: Retrospective cohort studies for identified signals

Retrospective cohort studies of three prioritised medication signals will be done, to quantify associations with adverse outcomes while adjusting for confounding. Details of the analysis plan will be determined by the specific drug and outcome being investigated.

### Timeline

There is no data collection for this project; data have already been extracted centrally by CPRD from routinely collected data. Record screening will be completed by June 2026. We anticipate generating results for objective 1 (descriptive analysis) by summer 2026; objective 2 (signal detection) by autumn 2026; objective 3 (prioritisation process) by spring 2027; and objective 4 (retrospective analyses) by summer 2027.

## Discussion

### Strengths

This study leverages contemporary large-scale, population-based electronic health records from national primary care databases, enabling assessment of prescribing during pregnancy across England. The use of validated pregnancy registers and established linkages to hospital admissions, mortality records, and socio-economic data strengthens outcome ascertainment and allows assessment of maternal as well as fetal and infant health.

A major strength is the systematic signal detection approach applied across all medications and a wide range of outcomes, rather than restricting analyses to pre-specified drug–outcome pairs. The use of advanced Bayesian and hierarchical methods allows information sharing across related medications and outcomes, improving the ability to detect potential safety signals, including for less commonly prescribed medicines.

The study team are a multi-disciplinary group with expertise in epidemiology, statistics, primary care, obstetrics, paediatrics, clinical pharmacology and lived experience of prescribing in pregnancy.

The integration of the quantitative findings with existing evidence and structured patient and expert input will enhance the relevance and transparency of the prioritisation process, and the prioritisation process will produce a valuable list of medications requiring further research in addition to the three that we will be able to investigate in this study.

### Limitations

As an observational study based on routinely collected data, there is potential for misclassification of exposures, outcomes, and of pregnancy status. The CPRD pregnancy register is based on an algorithm that searches for clinical codes likely to indicate pregnancy and generates estimated gestational dates from this. Some of the clinical codes used may generate false pregnancies, as has been highlighted in analyses of valproate use where the use of codes such as ‘*Advice on risk harm to fetus from maternl medictn dur preg’* are used when a patient is not pregnant [28]. To minimise the impact of this, where a pregnancy is based on a single code, the appropriateness of including these pregnancies will be reviewed based on the original codes used. Where HES linkage is available, we will also use this to triangulate whether a pregnancy is a true pregnancy, pregnancy outcome, and estimated gestation dates [17].

There is also the potential for residual confounding, particularly confounding by indication, which may not be possible to fully address through statistical adjustment. Prescription records indicate medications issued in primary care but we do not have data on whether prescriptions were collected and taken, so exposure misclassification must be considered.

Outcome misclassification is also possible due to imperfect coding, variation in recording practices, and limited clinical detail for some outcomes. Although signal detection methods are designed for hypothesis generation rather than causal inference, there remains a risk of identifying spurious associations arising from multiple testing or correlated exposures and outcomes. For these reasons, signals identified in this study will be interpreted cautiously and used primarily to prioritise medications for further investigation rather than to draw any definitive conclusions about causality.

### Dissemination

The results from previous similar cohort analyses, which enable adjustments for confounding due to morbidity, co-medications and other factors to be accounted for, have been judged to be of sufficient quality to influence regulatory decisions [29]. Therefore, if adverse outcomes are found to be associated with any of the three medications, the information will be presented to the Medicines and Healthcare products Regulatory Agency (MHRA) to feed into future regulatory decisions, directly benefitting pregnant women. The results will also be discussed with UKTIS (on our advisory board) to determine if any results should be added to bumps (best use of medicines in pregnancy) resources. These internet resources are widely used by clinicians and pregnant women, so any relevant study results can be used to help to improve the safety of pregnancy prescribing.

## Supporting information

S1 Table Grouping of medications based on BNF chapters and subsections. (DOCX)
