# Supplementary material for: Medicines prescribed in pregnancy: Protocol for a signal detection study using routinely collected data in England
Source: PLoS One. 2026 May 26;21(5):e0349045. doi: 10.1371/journal.pone.0349045 (PMC13210384; doi:10.1371/journal.pone.0349045)
Supplement: S1 Table — (DOCX) [file pone.0349045.s001.docx]

S1 Table: Grouping of medications based on BNF chapters and subsections

| Main Group | Subgroup |
| --- | --- |
|  |  |
| GASTROINTESTINAL | Dyspepsia, reflux & stomach protection |
|  | Laxatives |
|  | Other GI drugs |
|  | Anti-emetics |
|  |  |
| CARDIOVASCULAR | Beta blockers |
|  | Renin-angiotensin system drugs |
|  | Calcium channel blockers |
|  | Antiplatelets |
|  | Anticoagulants |
|  | Other cardiovascular |
|  |  |
| RESPIRATORY | Bronchodilators |
|  | Corticosteroids (inhaled) |
|  | Oral steroids |
|  | Leukotriene Receptor Antagonist |
|  | Antihistamines |
|  | Other respiratory |
|  |  |
| PSYCHIATRY & NEUROLOGY | Hypnotics & anxiolytics |
|  | Antipsychotics |
|  | TCA |
|  | SSRI |
|  | Other antidepressants |
|  | ADHD medication |
|  | Migraine treatment |
|  | Anti-epileptics |
|  | Smoking cessation |
|  | Other CNS |
|  |  |
| ANTI-INFECTIVES | Penicillins |
|  | Cephalosporins / beta-lactams |
|  | Tetracyclines |
|  | Macrolides |
|  | Sulphonamides & trimethoprim |
|  | Metronidazole, tinidazole & ornidazole |
|  | UTI antibiotics |
|  | Other antibiotics |
|  | Antifungals |
|  | Antivirals/antiprotozoals/antihelminitics |
|  | Vaginal & vulval infections |
|  |  |
| ENDOCRINE | Insulins |
|  | Metformin |
|  | Other oral diabetes medication |
|  | Thyroid hormones |
|  | Antithyroid drugs |
|  | Oral steroids |
|  | Female sex hormones & their modulators |
|  | Contraceptives |
|  | Other endocrine |
|  |  |
| HAEMATOLOGY / NUTRITION | Iron |
|  | B12 |
|  | Folate |
|  | Nutrition & blood |
|  |  |
| ANALGESIA | Paracetamol |
|  | Opioids |
|  | NSAIDs |
|  | Topical |
|  | Other |
|  |  |
| EYE, EAR & SKIN | Eye |
|  | Ear, Nose & Oropharynx |
|  | Skin - Topical |
|  | Skin - Oral |
|  |  |
| OTHER | Malignant disease & Immunosuppression |
|  | Genitourinary disorders |
|  | Allergen immunotherapy |

Other CNS (Drugs used in the treatment of obesity; Drugs used in parkinsonism and related disorders, Alcohol dependence, Opioid dependence, Drugs for dementia). Other antibiotics (Aminoglycosides; Clindamycin and lincomycin; Some other antibacterials). Other endocrine (Hypothalamic and pituitary hormones and anti, oestrogens; Drugs affecting bone metabolism; Other endocrine drugs)
